# Plant resilience: harnessing bioactive compounds for sustainable disease protection

**DOI:** 10.1007/s44154-026-00343-z

**Published:** 2026-10-26

**Authors:** Muhammad Zunair Latif, Miaomiao Deng, Naila Asghar, Lingguang Kong, Sumaira Kousar, Haimiao Zhang, Shams Ur Rehman, Yanke Jiang, Muhammad Humza, ChongChong Lu, Xinhua Ding

**Affiliations:** 1 State Key Laboratory of Wheat Improvement, College of Plant Protection, Shandong Agricultural University, Tai’an, 271018 China; 2 College of Agronomy, Shandong Agricultural University, Tai’an, Shandong 271018 China; 3 Department of Biochemistry, Government College Women University, Faisalabad, 38000 Pakistan; 4 State Key Laboratory of Wheat Improvement, Peking University Institute of Advanced Agricultural Sciences, Shandong Laboratory of Advanced Agriculture Sciences in Weifang, Weifang, Shandong 261325 China

**Keywords:** Plant resilience, Chemical defense, Phytohormones, Secondary metabolites, Antimicrobial peptides, Microbiome engineering

## Abstract

Plants have evolved complex phytochemical and immune defense mechanisms in response to pathogen invasion. Plants deploy phytochemical defense system—signaling molecules, secondary metabolites, antimicrobial proteins and peptides—to mitigate the pathogens they encounter. Pathogens have developed strategies—effector proteins, efflux pumps and detoxification mechanisms—to manipulate or overcome plant defenses. This review offers insights into how these bioactive compounds are biosynthesized and activated following pathogen recognition. We provide an overview of the dynamic crosstalk between PTI and ETI and their role in optimizing plant defense. Additionally, we discuss the potential of advanced genetic engineering approaches, nanocarrier-mediated elicitor delivery, and microbiome engineering to enhance these natural defenses. Finally, we address the technical bottlenecks of engineering plant immunity and provide a roadmap for future research directions. This review provides a stronger mechanistic foundation for breeding crops with durable, broad-spectrum resilience for sustainable crop protection.

## Introduction

Pests and plant disease outbreaks are a constant challenge to global agriculture and food security, with annual crop losses exceeding 20%–40% (Borrelli et al. 2021; Singh et al. 2023). Although traditional chemical disease control is promising, there is increasing concern regarding the emergence of pathogen resistance, environmental persistence and negative impacts on non-targeted organisms. Additionally, climate change is altering pathogen biogeography, host–pathogen interactions, and increasing disease outbreaks, leading to more frequent and unpredictable disease epidemics (Singh et al. 2023). To address these challenges, interest in understanding the plants innate defense mechanisms to specifically enhance plant resilience and prevent future disease outbreaks is increasing (Ristaino et al. 2021).

Classic plant pathology frames plant-pathogen interactions according to three interrelated but conceptually distinct paradigms: resistance, immunity and resilience. Resistance refers to the ability to prevent pathogen infection or restrict pathogen growth, whereas immunity refers to the molecular and cellular processes that underpin these defensive responses. A third concept is plant resilience, i.e., the ability to withstand or recover performance following perturbations, while maintaining essential physiological functions (Kissoudis et al. 2014; Tang et al. 2025). This resilience is supported by the complex plant immune system mediated by bioactive compounds. Plants and pathogens deploy parallel molecular tactics shaped by adaptive coevolution (Liu et al. 2026). Following pathogen perception, the plant immune system, consisting of pattern-triggered immunity (PTI) and effector-triggered immunity (ETI), is activated. Plants deploy phytochemical defense systems—signaling molecules, secondary metabolites, antimicrobial proteins and peptides—to fine tune immune responses (Jiang et al. 2025; Kanu and Kanu 2025; Somssich et al. 2025). Pathogens have developed strategies—effector proteins, efflux pumps and detoxification mechanisms—to manipulate or overcome plant defenses. In this review, we discuss the role of bioactive compounds in mediating immunity and their contribution to recovery and functional maintenance, the defining features of resilience. We provide an overview of plant immune system, focusing on chemical warfare and understanding the synthesis, regulation and deployment of bioactive compounds following pathogen recognition. We then evaluate advanced genetic engineering approaches to harness these bioactive compounds for increased resilience and describe the associated challenges. Finally, we outline key research questions and propose a strategic roadmap to guide future studies.

## Activation of the plant immune system: from recognition to generation of bioactive signals

### Two-tiered immune recognition system: PTI and ETI

Plants possess two-tiered immune system consisting of pattern-triggered immunity (PTI) and effector-triggered immunity (ETI) (Kanu and Kanu 2025). PTI is mediated by pattern recognition receptors (PRRs) that detect pathogen-associated molecular patterns (PAMPs) such as bacterial flagellin (flg22) or fungal chitin. This recognition activates signaling pathways including calcium influx, reactive oxygen species (ROS) bursts, and mitogen-activated protein kinase (MAPK) cascades, which leads to immune responses such as callose deposition (Wang et al. 2023; Weralupitiya et al. 2024). To counter PTI, pathogens secrete effector proteins (e.g., AvrPto in *Pseudomonas* spp.) that suppress host immunity. The second tier, ETI, is activated when intracellular nucleotide-binding leucine-rich repeat receptors (NLRs) recognize these effector proteins, triggering a hypersensitive response (HR) characterized by programmed cell death at the infection site (Huang et al. 2023; Spoel and Dong 2024). PTI and ETI function as highly interconnected signaling networks, forming a synergistic and amplified immune network. PTI acts as a broad-spectrum "baseline alert," whereas ETI functions as a "high-intensity alarm" triggered when specific threats are detected. The molecular basis of this synergy involves shared signaling hubs: 1) The EDS1-PAD4-ADR1 ternary complex serves as a central convergence point and is activated by both cell surface RLPs (e.g., RLP23) and intracellular NLRs (Pruitt et al. 2021). 2) Recognition of the fungal peptide nlp20 prompts RLP23 to recruit RLCKs (PBL30/31/32) via the adaptor SOBIR1, leading to ROS production, callose deposition, and MAPK phosphorylation, with the SOBIR1–PBL31 complex further enhancing PTI signaling (Böhm et al. 2014; Tian et al. 2021). 3) ETI-driven transcriptional reprogramming increases the expression of PTI-related genes. PTI and ETI can also trigger systemic acquired resistance (SAR) in distant tissues, resulting in broad-spectrum resistance against related or unrelated pathogens (Ding and Ding, 2020).

**Fig. 1:**
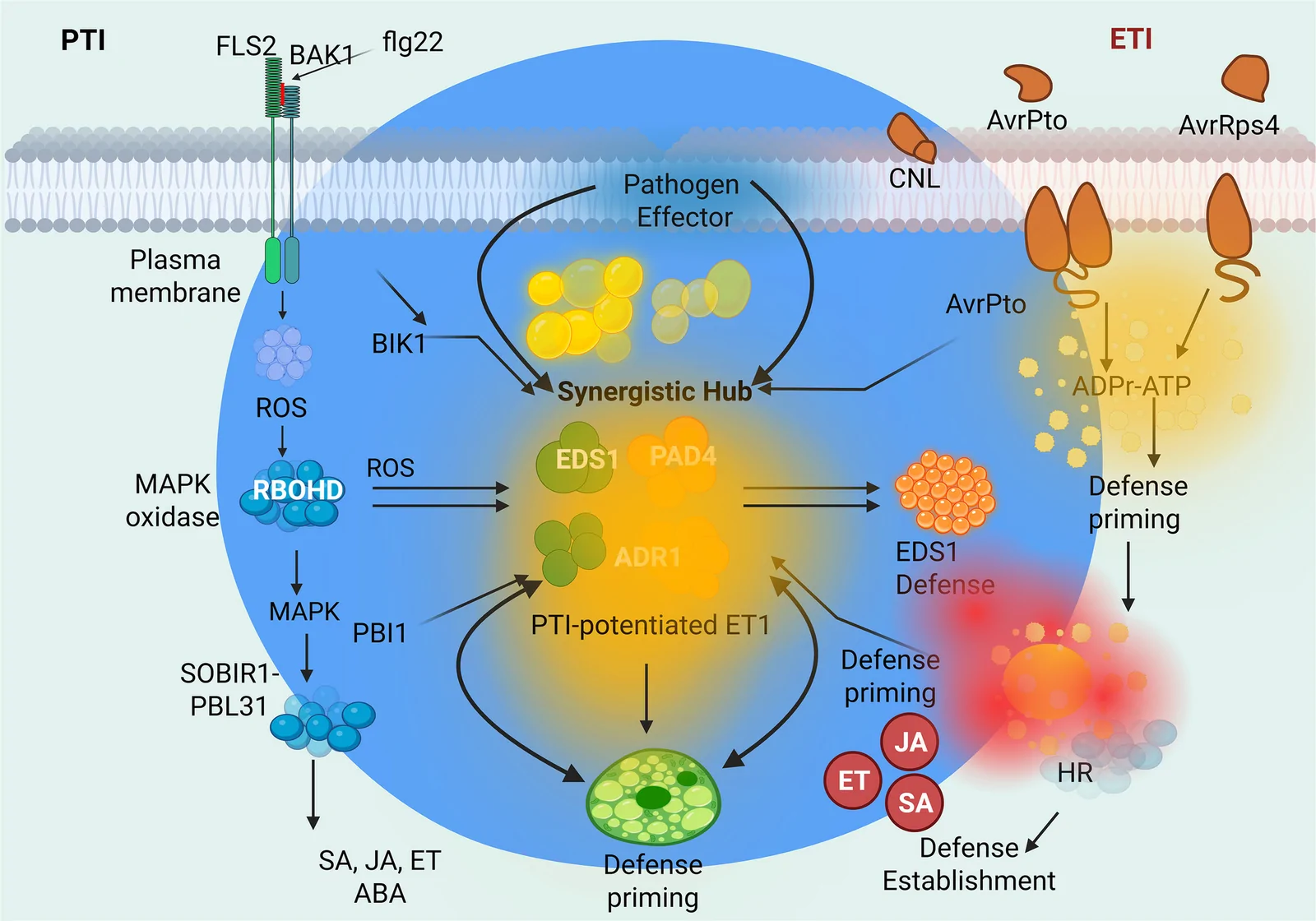
Schematic overview of co-existence of PTI and ETI as a function of immunity: Plants activate coordinated cascade immune responses following pathogen perception. PTI is activated at the infection site by MAMP (e.g., flg22) perception via cell-surface receptors (FLS2/BAK1), which leads to MAPK activation, Ca^2^⁺ influx, and ROS production via RBOHD. ETI is triggered by intracellular NLR receptors (e.g., CNLs) upon recognition of specific pathogen effectors. This synergistic crosstalk between PTI and ETI induces SAR and HR through the signaling molecules SA, JA, ET and ABA. The recognition of specific effector proteins activates a synergistic hub involving EDS1, PAD4 and ADR1, which are regulated by central regulatory hubs (EDS1-PAD4). This integration leads to transcriptional reprogramming, systemic signaling, and defense priming

Upon immune recognition, PTI and ETI synergistically trigger phytohormone signaling pathways that orchestrate local and systemic defense responses (Yu et al. 2024). These pathways converge on the shared salicylic acid (SA), jasmonic acid (JA), ethylene (ET) and abscisic acid (ABA) signaling hubs across multiple subcellular compartments, forming a pivotal regulatory interface that coordinates immune activation with whole-plant resilience (Fig. 1). The magnitude and hormonal balance of these signaling outputs collectively determine whether plants establish localized defense responses, systemic acquired resistance (SAR), or a coordinated resilience response that preserves growth while limiting pathogen spread.

#### Core bioactive signaling molecules: the central command of the hormonal network

Salicylic acid, jasmonic acid, and ethylene form the core hormonal network that regulates immune responses in plants. Abscisic acid, brassinosteroids (BRs), and gibberellins (GAs) act as modulators within this signaling network that integrate the defense response with plant growth, development and environmental adaptation (Wasternack and Song 2017; Tian et al. 2025). Plant immune regulation is therefore not controlled by a single hormone pathway but by a complex hormonal network in which signaling pathways interact positively or negatively depending on pathogen lifestyle, infection stage, and environmental conditions. Generally, SA-mediated signaling is associated with resistance against biotrophic and hemi-biotrophic pathogens, whereas JA and ET predominantly regulate defense against necrotrophic pathogens and herbivore insects. In contrast, ABA, BRs, and GAs perform more context‒dependent functions by adjusting the balance between immune activation and growth-related processes (Pieterse et al. 2014; Lozano-Durán and Zipfel 2015; Mishra et al. 2024; Tian et al. 2025). Overall, SA, JA, ET, ABA, BR, and GA constitute an interconnected hormonal network that tailors plant immune responses through pathogen-specific signaling and growth‒defense coordination. Understanding this network architecture is therefore essential for developing strategies to increase disease resistance without compromising crop productivity.

Salicylic acid plays key fundamental roles in different aspects of plant immunity, including PTI, ETI, SAR, induced systemic resistance (ISR), HR and cell death (Tian et al. 2025). There are two principal pathways for SA biosynthesis: the isochorismate synthase (ICS) pathway and the phenylalanine ammonia lyase (PAL) pathway (Zhu et al. 2025). In Arabidopsis and related Brassicaceae, SA is synthesized primarily via the chloroplast-localized ICS pathway, which accounts for more than 90% of defense-induced SA accumulation (Dempsey et al. 2011; Lefevere et al. 2020). In most terrestrial plants, such as rice, wheat, and tomato, the PAL pathway contributes substantially or primarily to defense-induced SA biosynthesis.

Recent discoveries have elucidated the PAL pathway, expanded this classical understanding of SA biosynthesis by revealing additional phenylalanine‒derived metabolic routes. Wang et al. (2025) identified a triple-enzyme module in rice comprising peroxisomal benzoyl-CoA:benzyl alcohol benzoyltransferase (BEBT), endoplasmic reticulum-associated cytochrome P450 benzylbenzoate hydroxylase (BBH), and cytosolic benzylsalicylate esterase (BSE), which sequentially converts benzoyl-CoA to benzylbenzoate, benzylsalicylate, and SA. Liu et al. (2025) characterized a parallel three-step enzymatic pathway that converts benzoyl-CoA into SA in *Nicotiana benthamiana*. This pathway involves BEBT, benzyl benzoate oxidase (BBO), and benzyl salicylate hydrolase (BSH), whose homologs are conserved across monocots and dicots. Zhu et al. (2025) provided a complete characterization through four functional SA-DEFICIENT GENES (OSD1-OSD4). OSD1 (cinnamoyl-CoA ligase) converts trans-cinnamic acid to cinnamoyl-CoA, which undergoes β-oxidation to benzoyl-CoA in peroxisomes. OSD2 (peroxisomal benoyltransferase) converts benzoyl-CoA to benzyl benzoate. OSD3 (endoplasmic reticulum cytochrome P450) hydroxylates benzyl benzoate to benzyl salicylate and OSD4 (cytoplasmic carboxylesterase) hydrolyzes benzyl salicylate to SA. These findings establish that the PAL pathway, rather than the ICS pathway, represents the primary route for SA biosynthesis in most crop species. A third minor route involving the malonic acid pathway has also been identified but it remains quantitatively insignificant across most species (Dempsey et al. 2011). This species-level diversity in SA biosynthesis pathways has important implications for engineering SA-mediated resilience, as enhancing PAL activity may be more effective than ICS manipulation in certain crops (Peng et al. 2021; Spoel and Dong 2024).

Once synthesized, SA acts as a key messenger that binds to the receptor protein NPR1. SA binding induces conformational remodeling of NPR1, releasing the C-terminal transactivation domain from its N‒terminal autoinhibitory BTB/POZ domain and promoting its transition from a cytoplasmic oligomer to a nuclear monomer (Ding et al. 2018; Zavaliev and Dong 2024. In the nucleus, the NPR1 protein assembles and interacts with TGA transcription factors to activate pathogenesis‒related (PR) genes and other defense‒associated transcriptional programs (Després et al. 2000; Zhou et al. 2000; Ding et al. 2018). The resulting PR proteins exhibit direct antimicrobial activity and prime distal tissues to respond more effectively to subsequent infections (Zribi et al. 2021). Although SA is essential for establishing SAR, SA itself does not appear to be the primary long distance mobile signal. Instead, SAR involves multiple mobile signals including methyl salicylate (MeSA), azelaic acid (AzA), glycerol-3-phosphate (G3P), dehydroabietinal (DA), and pipecolic acid (Pip), which collectively establish systemic priming in distal tissues (Park et al. 2007; Návarová et al. 2012). In addition, the role of SA in immunity also impacts wider cellular functions through crosstalk with JA, ET and ABA (Peng et al. 2021; Spoel and Dong 2024).

JA and ET represent another major immune signaling module that primarily regulates plant defenses against necrotrophic pathogens and insect herbivores (Zhang et al. 2025a). Following stress perception, JA biosynthesis is activated through the octadecanoid pathway, leading to the accumulation of bioactive JA derivatives that co-regulate the expression of downstream defense genes. JA signaling promotes the degradation of JAZ repressors and the activation of MYC transcription factors, resulting in increased production of secondary metabolites, such as alkaloids, glucosinolates, and terpenoids. ET signaling acts together with JA by activating EIN2/EIN3-dependent transcriptional responses that contribute to cell wall reinforcement, antimicrobial defense and pathogen containment (Pieterse et al. 2014). The interplay between the JA/ET and SA pathways often determines the specificity and effectiveness of plant immune responses. For example, antagonistic crosstalk between the SA and JA pathways ensures resource allocation to the most appropriate defense strategy on the basis of the type of pathogen attack (Nguyen et al. 2016). Moreover, ISR triggered by beneficial rhizosphere microbes largely depends on intact JA/ET signaling, allowing plants to establish defense priming for subsequent infection (Pieterse et al. 2014; Mashabela et al. 2022).

In addition to the SA-JA-ET signaling hub, ABA, BRs and GAs act as modulatory hormones that link immunity with growth and stress responses. ABA enhances resistance to stomatal-invading pathogens, such as *Pseudomonas syringae*, by inducing stomatal closure and limiting pathogen penetration. However, it can negatively regulate resistance to necrotrophic pathogens by suppressing JA-dependent defense pathways, increasing susceptibility to pathogens such as *Botrytis cinerea* and *Fusarium oxysporum* (Ton et al. 2009; Lozano-Durán and Zipfel 2015). This context-dependency highlights that ABA-mediated immune responses are largely determined by pathogen lifestyle rather than representing uniformly positive or negative defense regulators.

BRs contribute to plant immunity by integrating growth regulation with defense activation. BRs potentiate defense through positive modulation of JA and ET signaling by interacting with key transcription factors including BZR1 and HBI1, and can enhance defense through activation of MAPK cascades, ROS production, and immune gene expression (Lozano-Durán and Zipfel 2015). BR applications improve resistance against pathogens such as *Magnaporthe oryzae* in the rice and *P. syringae* in tomato, demonstrating its positive role in defense regulation. However, excessive BRs accumulation may increase susceptibility to certain viral pathogens, emphasizing the importance of maintaining balanced BR signaling for optimal defense responses (De Bruyne et al. 2014; Tian et al. 2025).

Gibberellic acid (GA) signaling provides another example of hormonal regulation of the growth‒immunity balance. GA generally suppresses SA-mediated defense through the degradation of DELLA proteins. However, DELLA proteins act as positive regulators of JA signaling. Therefore, GA-induced DELLA degradation can reduce the JA-dependent defense response (Blanco-Touriñán et al. 2020). Some pathogens such as *Gibberella fujikuroi* exploit this vulnerability by producing GAs to promote disease susceptibility. Conversely, GA-deficient mutants exhibit increased resistance to multiple pathogens (Blanco-Touriñán et al. 2020).

## Synthesis and deployment of bioactive defense arsenal synthesis and deployment of bioactive defense arsenal

### The “chemical arsenal” of secondary metabolites

**Fig. 2:**
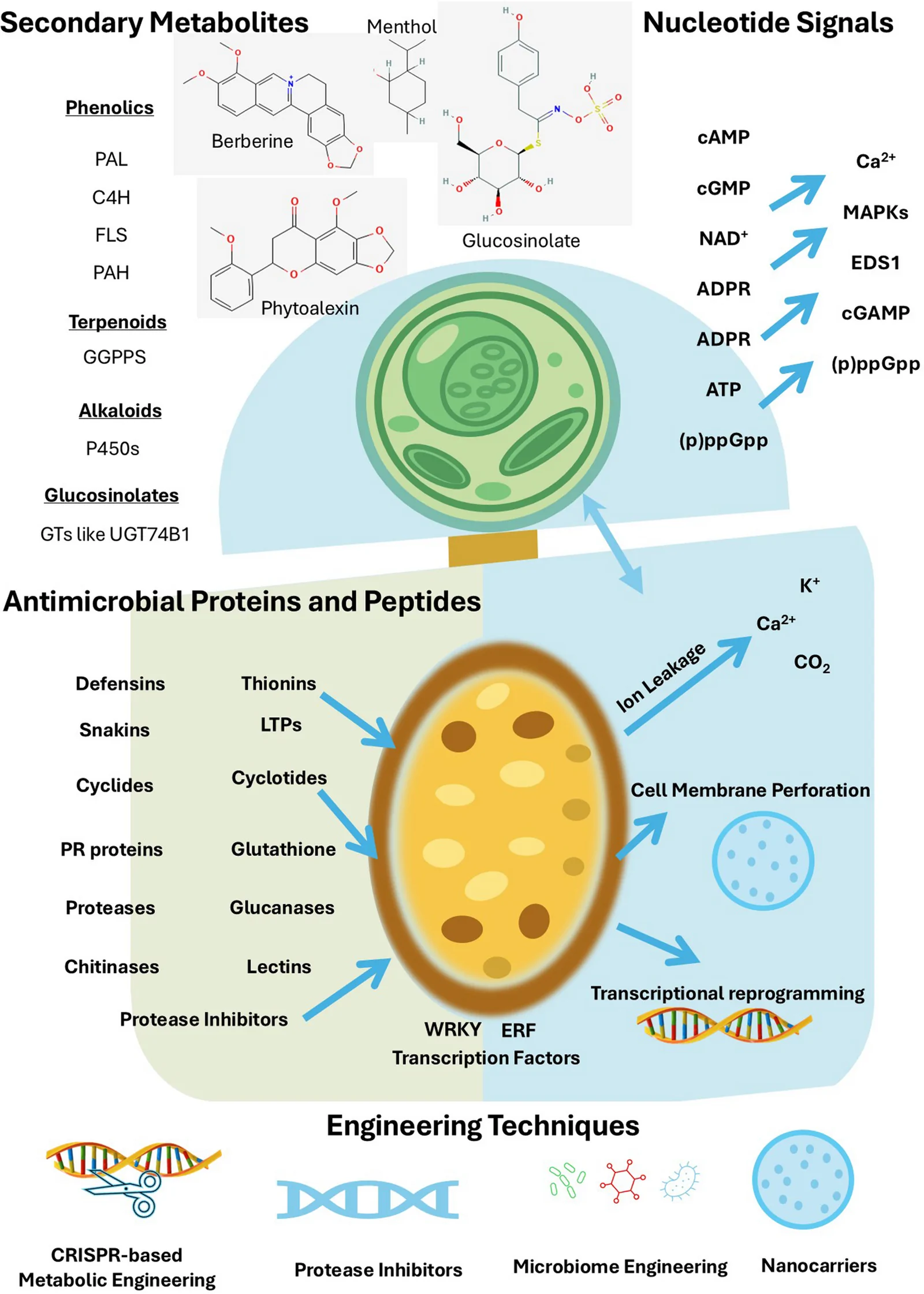
Integrated plant immune signaling and defense mechanisms: The schematic illustrates core biochemical pathways and components of induced plant defense. It includes secondary metabolites such as phenolic compounds, terpenoids, alkaloids and glucosinolates. Antimicrobial proteins and peptides including PRs, defensins, thionins, and cyclotides, act as direct pathogen inhibitors. Nucleotide second messengers (e.g., cAMP and cGAMP) and purine nucleotides (ATP, ADP) initiate signaling cascades involving Ca^2^⁺ flux and MAPK activation, which converge on key immune transducers such as EDS1. The figure also illustrates ion leakage, cell membrane perforation, and transcriptional reprogramming in response to microbial stress. Additionally, bioengineering approaches, such as CRISPR-mediated metabolic engineering, synthetic pathway design, microbiome engineering, and nanocarrier systems to increase natural immune responses and generate novel traits, are depicted

Plants have evolved a sophisticated chemical defense arsenal of secondary metabolites, antimicrobial proteins, peptides and nucleotide signals that are pivotal to the plant immune system (Fig. 2, Table 1). Secondary metabolites such as phenolic compounds, terpenoids, alkaloids, and glucosinolates act as natural defense agents. Antimicrobial proteins and peptides act as direct antimicrobial agents. Nucleotides and their derivatives have emerged as key signaling molecules in plant immunity.

**Table 1:** The plant bioactive defense arsenal: biosynthesis, mode of action, and engineering landscape

Compound Class	Biosynthetic Pathways and Key Enzymes	Primary Mechanisms of Action	Pathogen Counter-Strategies	Promising Engineering Approaches	References
Phenolics (Lignins, Flavonoids, Phytoalexins)	Phenylpropanoid pathway: *PAL* (Phenylalanine ammonia-lyase), *CHS/CHI* (Chalcone synthase/isomerase), *FLS* (Flavonol synthase)	Direct antimicrobial: Membrane disruption, enzyme inhibition. Structural: Lignin polymerization creates physical barriers. Signaling: ROS scavenging, modulation of auxin transport	Effector-mediated suppression of biosynthetic master regulators (e.g., MYB TFs). Secretion of detoxifying enzymes (e.g., laccases, peroxidases)	CRISPR-based activation of *PAL* and transcription factors Synthetic promoters for pathogen-inducible lignin deposition Heterologous expression of novel phytoalexin pathways	Ninkuu et al. 2022; Wang et al. 2022; Lv et al. 2024; Kaur et al. 2025
Terpenoids (Monoterpenes, Sesquiterpenes, Diterpenes)	MEP/MVA pathways: TPS (Terpene synthases), key diversification enzymes, GGPPS (Geranylgeranyl diphosphate synthase)	Direct antimicrobial: Membrane disintegration Volatile signaling: Priming defense in neighboring plants Anti-herbivory	Efflux pumps to reduce intracellular concentration Cytochrome P450-mediated oxidation and detoxification Modification of membrane composition for reduced permeability	Organelle engineering (chloroplast/mitochondria) to enhance precursor supply Pathway reconstruction in heterologous systems for production Engineered volatility for optimized aerial signaling	Cheng et al. 2025; El Arabi et al. 2025
Glucosinolates and Hydrolysis Products (e.g., Sulforaphane)	Amino acid-derived: CYP450s, GSTs (Glucosyltransferases)	Mustard Oil Bomb: Upon tissue damage, myrosinase-mediated hydrolysis to toxic isothiocyanates Broad-spectrum antimicrobial activity	Effector inhibition of myrosinase activity Secretion of glycosyl hydrolases to pre-emptively degrade precursors Behavioral avoidance of damaged tissues	Optimizing spatial separation of substrate and enzyme for controlled detonation Engineering hydrolysis thresholds to respond to specific pathogen elicitors Stacking different glucosinolate profiles for broader resistance	Sporer et al. 2021; Cheng et al. 2024
Antimicrobial Peptides (Defensins, Thionins)	Ribosomal Synthesis and Post-translational Modification	Membrane permeabilization: Cationic peptides disrupt microbial membrane integrity Pore formation leading to osmotic lysis Inhibition of cell wall synthesis	Protease secretion for peptide degradation Alteration of membrane charge (e.g., by adding lysyl-phosphatidylglycerol) to reduce binding Efflux pumps	Promoter engineering for strong, localized expression at infection sites Codon optimization and fusion proteins for enhanced stability and yield Design of synthetic peptides inspired by natural AMPs with enhanced potency	Jiang et al. 2025; Liu et al. 2025; Oliveira Júnior et al. 2025
Nucleotide-Based Signals (Nucleotides and Nucleotide derivatives)	Extracellular ATP (eATP): Synthesized via metabolic pathways cAMP and cGMP: Produced by adenylate cyclase and guanylate cyclase, respectively Hydrolysis of NAD(P)^+^ to various cyclic and linear derivatives (e.g., 2´-cADPR, ADRr-ATP) TIR-domain NADase activity of NLRs ppGpp: Synthesized by RelA-SpoT homologs (RSH) family proteins	eATP: Functions as a damage signal and activates early defense responses cAMP and cGMP: Act as second messengers in hormone signal transduction Hydrolysis of NAD(P)^+^ to various cyclic and linear derivatives (e.g., 2´-cADPR, ADRr-ATP) Involved in ETI immune response and systemic signaling ppGpp: Coordinates resource allocation and optimizes defense resource deployment	Depletion of substrates or precursors Secretion of hydrolytic enzymes to directly degrade nucleotide-based signaling molecules Inhibition of receptor perception by secreting effector proteins that interfere with the membrane localization or signal transduction capacity of eATP receptors Mimicry of second messengers to hijack downstream pathways	Engineer and overexpress eATP receptors to enhance initial sensitivity to damage and invasion Tissue-specifically enhance the synthesis of cAMP/cGMP at pathogen entry sites to precisely amplify early alarm signals Construct TIR-PRR chimeric receptors to create "synthetic NLRs," Induce the expression of ppGpp or its regulatory factors to prioritize resource allocation to defense when the plant is under stress	Witte and Herde 2024; Lu et al. 2026

Phenolics are multifaceted compounds with significant implications in mediating plant-defense mechanisms. They act as antioxidants and antimicrobial agents and regulate plant signaling pathways via hormonal crosstalk (Saini et al. 2024a, b). Most defense related phenolic (lignin and flavonoid) compounds are synthesized via the phenylpropanoid pathway. Lignin is a critical structural phenolic in pathogen defense, serving as a physical barrier that reinforces secondary cell walls and limits pathogen penetration (Tiwari et al. 2025). It is biosynthesized through the oxidative polymerization of three main types of monolignols (p-coumaryl alcohol, coniferyl alcohol and sinapyl alcohol) (Tiwari et al. 2025). Upon pathogen detection, plants initiate the transcriptional activation of genes (PAL, CCR, and CAD genes) responsible for lignin biosynthesis. These biosynthetic products drive rapid lignin deposition and the formation of callose-rich papillae at infection sites (Ma, 2024). In addition to structural defenses, lignin pathway intermediates such as coumarins, stilbenes and resveratrol function as phytoalexins that prevent pathogen colonization (Iqbal et al. 2024).

Flavonoids function as potent antimicrobial agents against bacterial and fungal pathogens by disrupting pathogen membranes and chelating divalent cations (Van-Acker et al. 1998; Oteiza et al. 2005). Flavonoid aglycones exhibit greater antibacterial activity than glycosylated derivatives do because of reduced steric hindrance (Waditzer and Bucar 2021; Han et al. 2026). Additionally, these compounds suppress bacterial efflux pumps, scavenge ROS, interfere with biofilm formation and nucleic acid biosynthesis, and modulate signal transduction pathways. These multi-target mechanisms make flavonoids less susceptible to pathogen resistance evolution than single-site synthetic pesticides (Cushnie and Lamb 2011; Ramaroson et al. 2022). Phytoalexins such as neodiosmin are specialized antimicrobial flavonoids produced in response to biotic or chemical stimuli that accumulate rapidly at infection sites (Lu et al. 2022). Rice momilactones, including momilactones A and B, accumulate upon *M. oryzae* challenge and exhibit potent antifungal activity. The core biosynthetic genes (CPS4, KSL4, CYP99A2/A3, MOMILACTONE A SYNTHASE) organize in a metabolic gene cluster on chromosome 4 (Shimura et al. 2007; Miyamoto et al. 2016). The genomic architecture of these genes makes the momilactone pathway a promising target for the metabolic engineering of enhanced blast resistance.

Terpenoids constitute the largest class of secondary metabolites, with diverse biological roles such as growth, defense and communication (Câmara et al. 2024; El Arabi et al. 2025). Terpenoid biosynthesis involves two primary pathways, the mevalonate (MVA) pathway and the methylerythritol phosphate (MEP) pathway, which serve as the precursors of mono-, di-, tri-, tetra- and sesquiterpenes (Câmara et al. 2024; El Arabi et al. 2025). Terpenoids such as limonene and resin acids act as natural insecticides, whereas farnesene inhibits spore germination and fungal growth (Gershenzon and Dudareva 2007; Zhou and Pichersky 2020). Many terpenoids, such as menthol and camphor, inhibit microbial growth by disrupting membrane integrity and interfere with ergosterol biosynthesis in *Candida* strains (Tholl 2015; Câmara et al. 2024). Volatile terpenoids mediate plant communication with neighboring organisms attracting natural predators and priming defense responses in nearby plants (Jin et al. 2025).

Alkaloids are nitrogen-containing organic compounds synthesized from amino acid precursors through complex biochemical pathways. Important pathways include the shikimate pathway and the mevalonate pathway (Facchini 2001; Saini et al. 2024b). These compounds exhibit broad-spectrum activities against pathogens and insect herbivores through multiple mechanisms of action, including membrane disruption, enzyme inhibition, and interference with protein synthesis. Alkaloids such as caffeine, nicotine, morphine and berberine are toxic to herbivores and pathogens (Saini et al. 2024b; Kumar et al. 2025).

Glucosinolates are secondary metabolites predominantly found in Brassicaceae. These compounds function as precursors to bioactive compounds that are activated upon tissue damage. These compounds are synthesized from amino acids such as methionine, tryptophan, and phenylalanine. The key enzymes in this pathway include cytochrome P450 enzymes and glucosyltransferases. Following pathogen attack or mechanical injury, these compounds are hydrolyzed by the enzyme myrosinase into bioactive products such as nitrile, isothiocyanates and thiocyanates. These compounds are involved in communication and induce a range of defense responses against pathogens and insect herbivore targets (Chhajed et al. 2020; Ting et al. 2020). The PEN2 myrosinase pathway, which uses the indole glucosinolate 4MI3G as a substrate, prevents fungal and oomycete entry at epidermal penetration sites, demonstrating spatial co-regulation with PTI responses (Bednarek et al. 2009).

### The “Precision Stike” of antimicrobial proteins and peptides

Antimicrobial proteins and peptides serve as core chemical weapons of the plant innate immune system enabling highly efficient and targeted action against pathogens (Farvardin et al. 2024; Yu et al. 2025) (Fig. 2). Plants secrete a range of defense proteins including PR proteins, protease inhibitors, glucanases, chitinases, lectins, and antimicrobial peptides. Chitinases and glucanases are hydrolytic enzymes that act as antifungals. Chitinases specifically hydrolyze β−1,4-glycosidic bonds in chitin, a major component of fungal cell walls, whereas β−1,3-glucanases break down glucan polymers. These enzymes act synergistically to disrupt the structural integrity of fungal cell walls, inhibit fungal growth, and may release PAMPs, such as chitin oligomers, which further activate plant immune responses as elicitors (Vaghela et al. 2022). Peroxidases reduce hydroperoxides (H_2_O_2_) or organic hydroperoxides produced during microbial attacks or in response to abiotic stresses. Proteases are crucial in signaling processes that activate the HR, effectively restricting pathogen spread (Freitas et al. 2024). Protease inhibitors can disrupt essential physiological processes by inhibiting proteases secreted by pathogens and insect herbivores (Moloi and Ngara 2023). Lectins contribute to pathogen recognition and modulate immune responses (Jain et al. 2022).

Antimicrobial peptides such as defensins, thionins and lipid transferases play essential roles in targeted defense. Defensins are cationic and cysteine-rich small peptides that exhibit broad-spectrum antimicrobial activities (Thevissen et al. 2004; Parisi et al. 2019; Fu et al. 2023). The mechanism of action of defensins involves initial binding to membrane phospholipids, followed by membrane permeabilization and the formation of pores. This process is facilitated by the amphipathic nature of defensin peptides, which allows them to insert into lipid bilayers and disrupt membrane integrity. Some defensins also exhibit additional mechanisms of action, including inhibition of cell wall synthesis, interference with protein synthesis, and binding to specific receptor proteins. The gene expression of defensins is tightly regulated by immune signaling pathways, which involve the recognition of PAMPs. This recognition triggers signaling cascades that activate transcription factors, leading to the expression of defensin genes (Contreras et al. 2020). These peptides initially bind to specific fungal membrane lipid rafts containing glucosylceramide (GlcCer), a lipid absent from plant membranes but essential for fungal growth. Binding triggers a conformational change that facilitates the insertion of the defensin's γ-core motif into the membrane, resulting in the formation of oligomeric pores of approximately 2–3 nm in diameter. This permeabilization leads to rapid efflux of K⁺ ions, dissipation of the membrane potential, and subsequent fungal cell death. In addition to membrane disruption, certain defensins (e.g., NaD1 from *Nicotiana alata*) enter fungal cells and bind to phosphatidic acid-enriched membranes of organelles, interfering with vacuolar fusion and triggering caspase-dependent apoptosis. The gene expression of defensins is tightly regulated by immune signaling pathways through WRKY transcription factors that bind to W-box elements in defensin promoters within 30–60 min of PAMP recognition (Contreras et al. 2020). Thionins, which are abundant in cereal grains, target pathogen cell membranes, leading to rapid pathogen cell death through osmotic lysis and triggering immune responses. They increase plant resilience by contributing to signaling pathways and modulating ROS production (Ha-Tran and Huang 2025; Riseh et al. 2025). Glutathione also plays a central role in plant–pathogen interactions by coordinating multiple mechanisms. These mechanisms include antioxidant defense, signal transduction, and metabolic reprogramming (Noctor et al. 2024).

### Nucleotide signals: novel messengers and regulatory networks in plant immunity

Nucleotides and their derivatives function as signaling molecules in plant immunity and stress adaptation, extending far beyond their traditional roles in central metabolism and nucleic acid synthesis. These molecules include extracellular ATP (eATP), extracellular nicotinamide adenine dinucleotide (eNAD⁺), cyclic nucleotides (such as cAMP, cGMP, and cADPR), and various metabolites derived from NAD⁺ degradation (e.g., ADP‒ribose, ADPR), among others (Fig. 2) (Witte and Herde 2024).

In recent years, breakthrough studies have continuously revealed the immune functions of novel nucleotide-derived signaling molecules. For example, eATP acts as a damage-associated molecular pattern (DAMP) that binds to the DORN1/P2K1 receptor, activating calcium signal transduction, ROS bursts, and the MAPK pathway, thereby serving as both a “danger alarm” and an “immune amplifier” in plant immunity (Chivasa et al. 2009; Matthus et al. 2020). Similarly, eCTP can also induce calcium influx and ROS production in plant cells, illustrating aspects of eATP signaling (Tanaka et al. 2010). Furthermore, 2′-deoxyguanosine (2-dG) functions as a potent immune elicitor that activates basal immune responses in plants (Lu et al. 2023).

The production of nucleotide signaling molecules constitutes a core step in their biological functions. The enzymatic activity of the Toll/Interleukin-1 Receptor (TIR) domain plays a central role in this process. Upon activation, TNL-type resistance proteins (Toll/Interleukin-1 receptor NLR proteins) primarily exert NAD⁺ hydrolase activity through their TIR domains, catalyzing the generation of diverse nucleotide-derived signaling molecules. These include ADP‒ribose (ADPR), ATP‒ADPR dinucleotide and its hydrolytic products, as well as various cyclic ADPR variants, such as cADPR, 2´-cADPR, and 3´-cADPR. These signaling molecules subsequently activate downstream helper NLR proteins (e.g., NRG1), promoting the assembly of channel-forming resistosomes and thereby triggering a strong immune response (Yu et al. 2022; Kwiatkowski et al. 2024; Song et al. 2024). Studies have further revealed that activated TNLs produce a novel signaling molecule termed ADRr-ATP. This molecule specifically binds to the EDS1–SAG101 complex, inducing a conformational change in SAG101 that enhances the interaction between EDS1–SAG101 and the helper protein NRG1, leading to the formation of a stable EDS1–SAG101–NRG1 ternary complex. These complexes undergo oligomerization and form calcium-permeable channels on the plasma membrane. The resulting calcium influx ultimately triggers robust immune responses such as HR (Xiao et al. 2025). This sophisticated nucleotide signaling landscape plays a central role in immune activation and stress adaptation, suggesting novel targets for engineering plant resistance.

## Pathogen counterstrategies: the evolutionary arms race

**Fig. 3:**
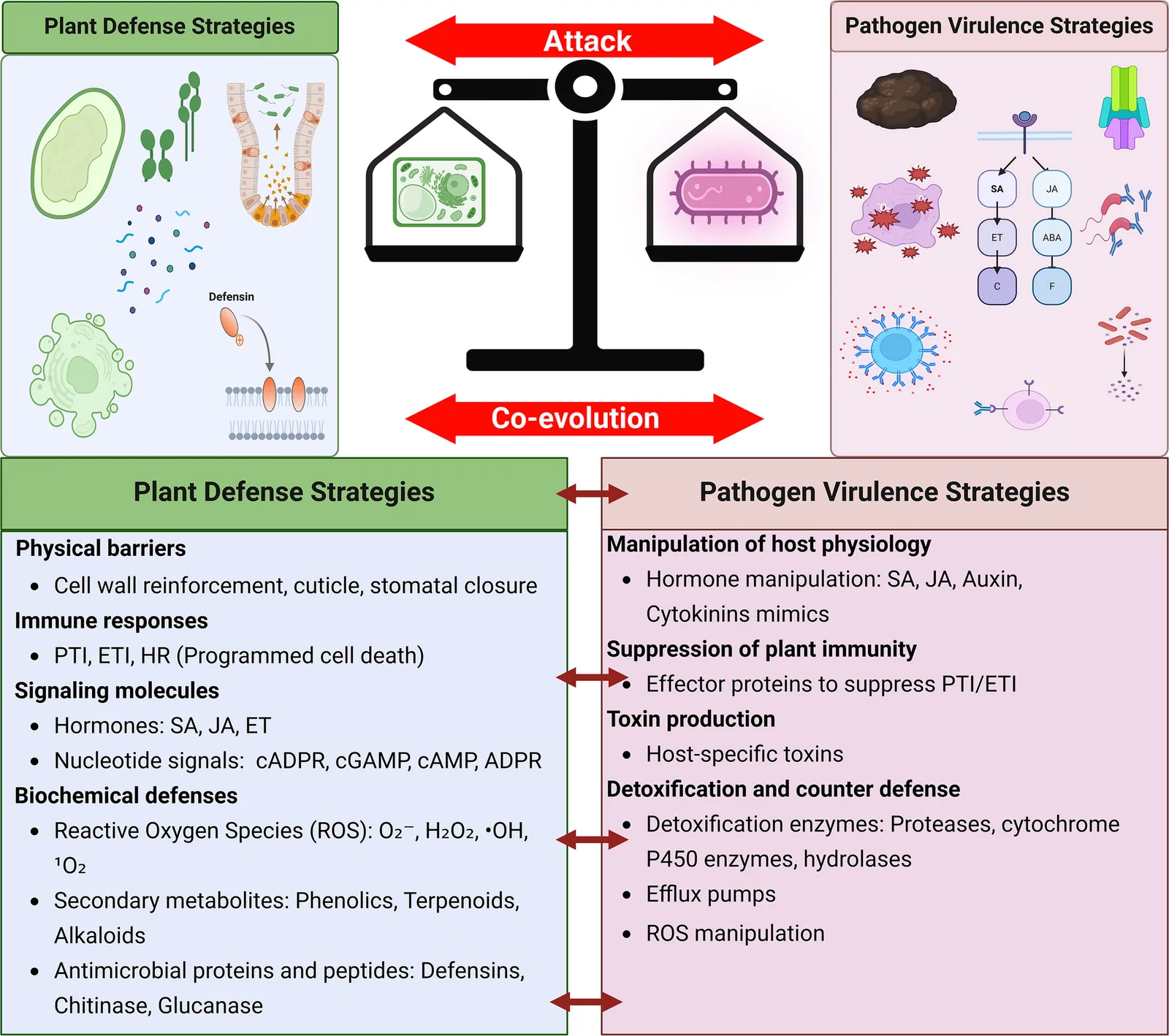
Co-evolution of plant defense and pathogen virulence mechanisms: A diagram summarizing the ongoing evolutionary battle between plant defense and pathogen virulence mechanisms. The left panel highlights plant defense strategies, whereas the right panel highlights pathogen virulence mechanisms to overcome plant defense barriers. The central arrows represent the continuous cycle of attack and counterattack, resulting in co-evolution

Each of the pathogen counter strategies described below directly targets a specific layer of plant immunity introduced. Effector proteins suppress PTI by interfering with PRR complexes or MAPK cascades, and they manipulate phytohormonal networks by mimicking or antagonizing SA, JA, and ET signaling. Detoxification mechanisms neutralize the secondary metabolites and antimicrobial peptides that plants deploy as chemical weapons. ROS-manipulation strategies counteract the oxidative burst generated during PTI, whereas physical barriers such as melanin protect pathogens from AMPs. This one-to-one correspondence is illustrated in Fig. 3, where left-panel plant defenses align horizontally with right-panel pathogen counter strategies.

### Effector interference strategies: sabotaging immune perception and signaling

#### Targeting perception/signaling

Pathogens subvert immune responses by developing effector proteins that target various components of plant defense systems (Fig. 3). These effectors inhibit host metabolic processes, defense mechanisms, and signal transduction pathways to promote susceptibility (Hogenhout et al. 2009; Bauters et al. 2021; Wang et al. 2022; Zhang et al. 2026). Bacterial effectors target early steps in PTI signaling, including the inhibition of MAPK cascades, disruption of protein‒protein interactions, and interference with calcium signaling. For example, the *P. syringae* effector AvrPto directly interacts with PRR complexes, inhibits their kinase activity, and prevents the activation of downstream signaling pathways. Fungal and oomycete effectors often target transcriptional regulators and other nuclear processes to subvert transcriptional reprogramming (Kale 2012; Kim et al. 2020). The *P. infestans* effector Pi03192 suppresses host cell death by targeting an NAC transcription factor (McLellan et al. 2013), whereas the *Magnaporthe oryzae* effector Avr-Piz-t interferes with the function of the E3 ubiquitin ligases APIP6 and OsATL32 (Park et al. 2012; Yan et al. 2025). The suppression of PTI allows pathogens to establish infection and proliferate within host tissues (Martin 2011).

#### Hormone manipulation

Pathogens manipulate phytohormone signaling to evade specific immune responses and facilitate their dissemination. A key strategy exploits the mutual antagonism between SA and JA pathways (Ma and Ma 2016; Borrelli et al. 2021). In addition to cell surface signaling, many pathogens produce hormone analogs or effectors that interfere with phytohormone biosynthesis or signaling. Mutual antagonism between the SA and JA pathways is a classic example of the hijacking of hormonal crosstalk. The SA pathway often confers resistance to biotrophic pathogens, which attenuates JA signaling, thereby compromising resistance to necrotrophic pathogens. The activation of the JA pathway enhances resistance to some necrotrophic pathogens but inhibits the SA pathway and resistance to biotrophic pathogens (Kazan and Lyons 2014). For example, *B. cinerea* produces an exopolysaccharide that activates SA signaling via a tomato NPR1 homolog, thereby suppressing antagonistic JA signaling (El Oirdi et al. 2011). Conversely, the biotroph *P. syringae* produces coronatine, which mimics JA and suppresses SA-mediated defenses (Kazan and Lyons 2014). In addition to SA‒JA crosstalk, pathogens also manipulate other hormone pathways to increase susceptibility. For example, *Ustilago maydis* secrete chorismate mutase (Cmu1) into host cells to interfere with the SA signaling pathway in maize (Djamei et al. 2011). Similarly, *P. syringae* T3E AvrRpt2 increases auxin levels during infection (Llorente et al. 2008; Fu et al. 2011). The manipulation of ethylene signaling is another target for pathogens that alter host responses and provide pathogens with opportunities to target weakened host defenses.

#### Inhibition of defense compound biosynthesis and perception

Some effectors can directly disrupt the biosynthesis of antimicrobial compounds. For example, the *Xanthomonas* effector XopM targets the allene oxide synthase OsAOS3, which interferes with the JA pathway and modulates jasmonate-mediated defense in rice (Li et al. 2024). Alternatively, pathogens secrete enzymes that degrade antimicrobials post-biosynthesis. Many pathogenic fungi suppress phytoalexin biosynthesis by producing enzymes that degrade specific phytoalexins or their precursors, allowing them to survive in the presence of high concentrations of antimicrobial compounds (Pedras and Abdoli 2017; Westrick et al. 2021). Some pathogens can interfere with the transport and accumulation of antimicrobial compounds at infection sites. This may involve disrupting transporter proteins, altering the cellular pH, or producing compounds that interfere with antimicrobial agents.

### Detoxification and tolerance mechanisms: neutralizing and resisting antimicrobials

#### Detoxification

Pathogens employ enzymatic detoxification, efflux pumps, and physical tolerance mechanisms to survive host chemical defenses (Fig. 3) (Wang et al. 2022). Some pathogens produce proteases that directly target antimicrobial peptides and cleave them into inactive fragments (Sabbadin et al. 2021). Fungal pathogens such as *Sclerotinia sclerotiorum* produce cytochrome P450 enzymes to detoxify phytoalexins into inactive derivatives (Pedras and Abdoli 2017). Others secrete specific hydrolases; *Cladosporium fulvum* secretes tomatinases that convert α-tomatine into less toxic derivatives (Ökmen et al. 2013), and *Fusarium graminearum* secretes glycosyl hydrolases to detoxify saponins and colonize their host plants (You et al. 2024). Bacterial pathogens often possess a plethora of efflux pumps that can expel a wide range of antimicrobial agents from the cell. This reduces intracellular concentrations to subtoxic levels, allowing them to resist, survive, and escape host defense (Laborda et al. 2024). These pumps are specific to different classes of compounds and can be induced by exposure to antimicrobial molecules.

#### ROS manipulation

Pathogens have evolved sophisticated antioxidant systems to detoxify or scavenge ROS (Park and Son 2024). These systems can be categorized into enzymatic (catalases, glutathione peroxidases, and superoxide dismutases) and nonenzymatic (glutathione, ascorbic acid, and carotenoids) antioxidants, which work together to scavenge ROS (Santos-Sánchez et al. 2019; García-Caparrós et al. 2021). Some pathogens secrete effectors that suppress ROS production or detoxify ROS. For example, the bacterial effector AvrPtoB inhibits NADPH oxidase activity, reducing the oxidative burst and promoting pathogen virulence (Xiang et al. 2008). Bacterial pathogens such as *Pseudomonas* secrete extracellular polysaccharides to form a protective layer against oxidative stress (Fones and Preston 2012). Some pathogens produce ROS-scavenging compounds that can neutralize host-derived oxidative stress, whereas others have evolved mechanisms to prevent the accumulation of ROS. The protein Pep1 interferes with plant peroxidase activity. This interference with host-derived ROS production represents a proactive strategy for avoiding oxidative damage (Dong et al. 2011; Hemetsberger et al. 2012). Some pathogens exploit ROS to promote infection. For example, *B. cinerea* produces ROS to induce host cell death and facilitate tissue colonization (Govrin and Levine 2000).

#### Physical and structural protection mechanisms

Pathogens also employ physical barriers to resist antimicrobial agents. For example, many fungi produce melanin, and the porous architecture of melanin protects against oxidative stress and acts as a barrier to host defense proteins (Fig. 3) (Nosanchuk et al. 2015; Eisenman et al. 2020). Melanin biosynthesis is often induced by exposure to plant defense compounds, suggesting its role as an adaptive response to host defenses. The regulation of melanin production involves multiple signaling pathways that integrate information about environmental stress and antimicrobial exposure (Jia et al. 2025). Another tolerance strategy is the modification of cellular structures. Some pathogens alter their membrane composition to reduce permeability, thus preventing the entry of toxic compounds in response to plant defense compounds. These membrane modifications can include changes in lipid composition, the incorporation of protective compounds, and alterations in membrane protein expression (Willdigg and Helmann 2021). Others protect their cell walls from lysis and detection by the host through convergent evolution (Rovenich et al. 2016).

## Engineering strategies for enhanced resilience: modulating bioactive responses

### Precision genome engineering for reprogrammed immune recognition and signaling

Advanced genetic engineering and genome editing technologies have transformed our ability to modify the plant immune system precisely. These precision tools enable direct editing of genes that control pathogen detection, defense signaling, and the production of antimicrobial compounds. Such approaches accelerate the development of crops with durable and broad-spectrum resistance. Among them, the emergence of the CRISPR-Cas9-based system has been particularly transformative, enabling precise disruption of susceptibility (S) genes, functional enhancement of resistance (R) genes, and fine-tuning of defense-associated signaling cascades (Table 2) (Chattopadhyay et al. 2025; Tuncel et al. 2025).

**Table 2:** Strategic framework for engineering plant immunity: comparative analysis of advanced technologies

Technology	Core Applications in Immunity	Key Mechanistic Strengths	Primary Limitations and Biological Challenges	References
Precision Genome Editing (e.g., CRISPR-Cas)	NLR/PRR engineering and stacking Susceptibility (S) gene knockout Fine-tuning hormone signaling nodes (e.g., JAZ, NPR1) Metabolic pathway optimization	High precision and multiplex capability Enables transgene-free outcomes Direct manipulation of native loci, accelerating trait introgression	Off-target effects in complex genomes Variable delivery efficiency across crops Regulatory hurdles for edited crops Pleiotropic effects from perturbing networked genes	Ngou et al. 2025; Yang et al. 2025; Venkataraman et al. 2025
Nanocarrier Delivery Systems	Targeted delivery of elicitors (e.g., SA, RNAi) Foliar micronutrient supply (e.g., Si, Zn) Integration with biosensors for precision release	Enhanced stability and cellular uptake of biologics Spatio-temporal control via targeted (e.g., stomatal) delivery Multi-component co-delivery to activate synergistic pathways	Scalability and cost-effective manufacturing Unclear long-term ecotoxicology profile Potential for nanoparticle-induced oxidative stress in plants	Kohay et al. 2024; Pagano et al. 2025; Puangpathumanond et al. 2025
Microbiome Engineering	Induced Systemic Resistance (ISR) priming Pathogen exclusion via niche competition Mobilization of bioavailable nutrients (e.g., Fe, P)	Self-amplifying and environmentally sustainable Provides broad-spectrum, multi-pathogen protection Leverages pre-evolved, beneficial plant–microbe interactions	Establishment efficacy and consistency across field environments Host genotype-dependent success Potential for unintended ecological shifts	Ali et al. 2024; Zhou et al. 2024; Dubey et al. 2025
Synthetic Biology Circuits	Pathogen-inducible promoter-driven defense genes Logic-gated responses for tissue-specific activation Feedback-controlled expression to minimize fitness costs	Minimal fitness cost due to precise temporal control Programmable and orthogonal signaling pathways Potential for novel immune outputs not found in nature	Context-dependent performance and biological "noise" Immense complexity of predictive design in whole plants Regulatory and public acceptance hurdles for GMOs	Khan and Lister 2025; Kong et al. 2025; Lloyd et al. 2025
Advanced Breeding Analytics (Genomic Selection, Speed Breeding)	Pyramiding quantitative trait loci (QTL) for durable resistance. Accelerated introgression of wild allele variants (e.g., *Rpv3*) Haplotype-based selection for complex traits	To precisely and efficiently mine and pyramid optimal genetic resources Enable the direct evolution of complex disease resistance traits Breed elite cultivars with durable and broad-spectrum resistance	Accuracy and environment-dependency of QTL identification Large breeding scale and selection pressure. Genetic incompatibility High complexity of haplotype identification and analysis	Nannuru et al. 2025; Escamilla et al. 2025

Engineering strategies increasingly focus on reprogramming PRRs and NLRs. Extracellular immunity can be broadened by transfer of PRRs across plant families. For example, the introduction of the *A. thaliana* EFR receptor, which recognizes the bacterial elongation factor Tu, into tomato and solanaceous plants confers resistance to multiple bacterial pathogens (Lacombe et al. 2010; Piazza et al. 2021). Reverse engineering of natural PRR variants such as VrFLS2XL and GmFLS2b has revealed strategies to optimize receptor‒ligand binding and strengthen co-receptor interactions, expanding recognition to include evasive PAMPs (Zhang et al. 2025b). The Arabidopsis RLP23 receptor, when introduced into tomato, rice, and poplar, confers broad-spectrum resistance, which is further strengthened by engineering its intracellular domain (Yang et al. 2025). Moreover, receptors such as RXEG1 in soybean and cotton have the potential to target conserved microbial enzymes for wide-ranging resistance (Zeng et al. 2025).

For intracellular immunity, innovative decoy engineering has emerged as a powerful approach. The incorporation of a heavy metal-associated (HMA) domain into the rice NLR Pik enables the recognition of multiple effectors from *M. oryzae*, resulting in durable resistance (Maidment et al. 2021). A similar strategy was applied to Arabidopsis PBS1, where replacement of its cleavage site with protease-recognition motifs increased the recognition specificity of the RPS5 NLR. In parallel, stacking multiple NLRs with complementary recognition specificities into a single locus creates a polyvalent receptor system that is significantly more challenging for pathogens to bypass. CRISPR-Cas9 now facilitates direct editing of native PRR and NLR alleles, accelerating the translation of engineered immune traits from laboratory studies to field applications.

The defense hormone network is another critical target for genetic modification, although these pathways are often subject to fitness trade-offs. Precision engineering offers opportunities to fine-tune this balance. For example, the overexpression of the positive regulator NPR1 enhances resistance in wheat and rice without severe pleiotropic effects, particularly when its translation is driven by pathogen-responsive upstream open reading frames (uORFs_TBF1_) of the *TBF1* gene (Xu et al. 2017). Conversely, CRISPR‒Cas9-mediated knockout of negative regulators, such as JAZ repressors, leads to constitutive JA pathway activation and improved resistance to necrotrophic pathogens. Similarly, targeting negative regulators of the SA pathway or central nodes in growth–defense trade-offs, such as DELLA proteins or specific AP2/ERF transcription factors, can strengthen immunity while minimizing yield penalties.

Another promising strategy is the metabolic reprogramming of secondary metabolite pathways that produce bioactive compounds. The overexpression of rate-limiting enzymes, such as stilbene synthase, in grapevines enhances the synthesis of the antimicrobial resveratrol and resistance to powdery mildew (Liu et al. 2019). CRISPR‒Cas9 further facilitates these efforts by enabling the deletion of pathway repressors or by fine-tuning promoter elements to increase the expression of biosynthetic genes in response to infection, yielding plants with inducible, high-potency metabolic arsenals (Tuncel et al. 2025). Notably, the simultaneous overexpression of key enzymes, such as phenylalanine ammonia-lyase (PAL) or terpene synthases, has successfully increased the production of phenolic compounds and antimicrobial terpenoids in various crops, although metabolic bottlenecks or intermediate toxicity remain significant challenges.

Synthetic biology offers transformative potential through the design of programmable defense circuits. This includes engineering synthetic promoters that activate specifically upon pathogen detection, minimizing metabolic cost, and devising artificial antimicrobial compounds with tailored activity and specificity (Belmonte 2025). Recent progress in multi-gene expression has further represented a significant advancement in metabolic engineering, enabling the coordinated activation of defense modules.

### Nanobiotechnology: precision tools for targeted delivery and immune priming

Nanobiotechnology is reshaping plant disease management through the development of precision tools that operate at the nanoscale. These approaches address the limitations of conventional methods, including non-target effects, pesticide resistance, and delayed diagnostics. Key mechanisms such as targeted delivery, enhanced biocompatibility, and the ability to induce systemic plant responses position nanobiotechnology as a transformative force in modern phytopathology (Table 2) (Sangwan and Singh 2025; Lu et al. 2026).

Nanoparticles (NPs) function as antimicrobial and defense elicitors. Engineered NPs such as silicon and chitosan stimulate redox signaling, activate SA-dependent defense pathways, and increase antioxidant enzyme activity. For example, silicon NPs interact with apoplastic proteins to increase intracellular signaling, whereas foliar application of chitosan NPs increases the expression of defense-related genes and increases the phenolic content (Chandra et al. 2015; Miao et al. 2025). In Nano-priming, seed treatment with NPs such as silicon dioxide or chitosan enhances stress resistance and activates SAR. This process increases the expression of defense-related genes and antioxidant activity, resulting in improved germination, vigor, and seedling health (Chandra et al. 2015). Metallic and metal oxide NPs, including silver, copper, zinc, titanium dioxide, and magnesium oxide, exhibit broad-spectrum activity against phytopathogens through mechanisms such as membrane disruption, ROS generation, and interference with cellular processes. Compared with conventional single-site inhibitors, their multi-target mode of action reduces the likelihood of resistance development (Kamyab et al. 2023). Metallic NPs, including silver, zinc, and silicon, additionally serve as micronutrient sources and stress cues.

Nanocarrier systems, including liposomal, polymeric, and mesoporous silica NPs, enable efficient and controlled delivery of active compounds such as fungicides, RNA interference (RNAi) molecules, or defense elicitors (Santana et al. 2020; Sangwan and Singh 2025). These carriers increase compound stability, promote cellular uptake, and allow targeted release at infection sites, thereby increasing compound efficacy while minimizing off-target effects (Liu et al. 2024; Sangwan and Singh 2025). Surface-functionalized NPs facilitate stomatal delivery and organosilica systems for co-delivery of immune-priming compounds. For example, a surface ligand-engineered NPs system (SENDS) was developed for targeted delivery to stomata. Compared with non-target NCs, foliar application of SENDS disrupted *X. campestris* invasion, enhanced plant defense, and reduced pathogen growth by 20-fold without impairing natural stomatal function (Puangpathumanond et al. 2025). Other advances include amidase- and redox-responsive nano-delivery systems, in which SA is linked with functional disulfide-doped mesoporous SiNPs to co-deliver SA and bioavailable Si to stomatal tissues in rice leaves, effectively inducing SAR and ISR against sheath blight (Liang et al. 2025). Mesoporous organosilica NCs have also been employed for the controlled release of SA and cymoxanil, achieving 86.22% control efficiency against cucumber downy mildew and activating immune responses by regulating resistance-related genes (Zhang et al. 2024).

Nano sensor technology enables real-time, in-field pathogen detection through the integration of nanomaterials such as gold NPs, quantum dots, and carbon nanotubes. These sensors convert biological recognition events such as binding to pathogen-specific biomarkers or volatile organic compounds into quantifiable signals, permitting early diagnosis and intervention (Shaw and Honeychurch 2022). Nanotechnology also offers new tools for genetic manipulation and RNA interference–based disease management. Among the most promising developments is nano-enabled gene delivery, which employs clay-based or carbon nanomaterials to protect and transport double-stranded RNA into plant tissues. This approach, exemplified by BioClay™ technology, induces sequence-specific silencing of essential pathogen genes without genetic modification of the host plant, which confers durable resistance against viral and fungal pathogens (Kandhol et al. 2025).

### Advanced breeding integrated with microbiome management

Advanced breeding strategies are essential for unlocking complex, quantitative disease resistance traits (Table 2). Marker-assisted selection (MAS) enables the introgression of broad-spectrum resistance genes into elite cultivars. For example, the addition of blast resistance genes (Pi2, Pi9, and Piz-t) into elite rice lines significantly increased durability against *M. oryzae* (Ashkani et al. 2015). The efficacy of MAS depends on validated marker‒trait associations, enhanced by high-throughput genotyping technologies (Hasan et al. 2021). For complex polygenic traits such as Fusarium head blight (FHB) resistance in wheat, genomic selection (GS) outperforms phenotypic selection by accelerating the development of lines with stable resistance from multiple small-effect QTLs (Budhlakoti et al. 2022). Natural variation within germplasm collections underpins both MAS and GS, as exemplified by the Rpv3 locus conferring downy mildew resistance in wild grapevine, which is introgressed into commercial cultivars via MAS (Foria et al. 2020).

Complementing these genetic strategies are microbiome interventions designed to prime the plant's immune system (Table 2). This approach harnesses beneficial microbes that act as natural immunizers. The integration of microbiome management with advanced breeding represents an emerging strategy for holobiont-based resilience. Breeding programs can select root traits that favor the establishment of beneficial rhizosphere microbiota. Plant growth-promoting rhizobacteria (PGPRs), such as *P. fluorescens* WCS417r, induce systemic resistance in Arabidopsis, tomato, and rice against multiple pathogens via JA and ethylene signaling (Pieterse et al. 2014). Similarly, plant growth-promoting fungi (PGPFs), such as *Trichoderma asperellum* T34, prime defense responses in tomato and cucumber against *B. cinerea* and *Pythium ultimum* by increasing the activity of defense-related enzymes and the expression of PR genes (Hermosa et al. 2013). A specific example of successful integration involves the genotype-dependent recruitment of beneficial rhizobacteria through the root exudate composition. The plant genotype has been shown to significantly influence the colonization capacity of *Pseudomonas fluorescens* SBW25 in wheat, with cultivar identity determining both the abundance and community structure of root-associated microbial populations (Guan et al. 2024). Root-secreted malic acid recruits beneficial soil bacteria to the rhizosphere, activating systemic resistance pathways (Rudrappa et al. 2008). At the molecular level, MYB72-dependent coumarin exudation in Arabidopsis selectively affects the composition of the root microbiome by favoring the recruitment of beneficial taxa over that of pathogenic ones (Stringlis et al. 2018).

In parallel with the use of living microbes, synthetic elicitors are applied. Benzothiadiazole (BTH), a functional analog of SA, is a landmark success. It is commercially registered as Actigard or Bion and activates SAR in wheat, tobacco, and melons against bacterial, fungal, and viral pathogens (Görlach et al. 1996). The future of applying these agents lies in precision agriculture, where they are deployed only in response to real-time pathogen pressure detected by advanced sensors. This targeted approach minimizes resource use and metabolic burden on crops while maximizing protection efficacy, representing a sophisticated and sustainable strategy for modern crop management.

## Challenges and future perspectives in engineering plant immunity

### Biological complexities and trade-offs

Although the molecular arms race between plants and pathogens is a primary driver of evolution, the deployment of engineered resistance traits must be considered within a broader ecological and evolutionary context. The engineering of plants for increased defense and resilience can have significant pleiotropic effects. A primary constraint is the inherent fitness cost associated with defense activation (Gao et al. 2024). Plants must allocate limited resources between growth, defense, and reproduction to maintain the right balance. Excessive investment in any one area can potentially compromise others. The manipulation of bioactive compound production can also have pleiotropic effects on plant physiology and ecological interactions. Overproduction of SA can increase resistance toward pathogens but also has negative impacts on plant growth, mycorrhizal associations and the colonization of beneficial bacteria (Pokotylo et al. 2022; Ortega et al. 2024). Elevated levels of JA can increase resistance to necrotrophic pathogens and insects but may also reduce plant growth, as observed in *A. thaliana* and *Nicotiana attenuate* (Heinrich et al. 2013). The overexpression of flavonoid biosynthesis genes in potato tubers increased the antioxidant capacity but significantly decreased the starch content in transgenic potato plants (Lukaszewicz and Szopa 2005). Increased emissions of herbivore-induced plant volatiles (HIPVs), such as specific terpenoids, can increase indirect defenses by attracting predators and parasitoids of herbivores (Huang and Dudareva 2023; Ramya et al. 2025). However, these same signals can inadvertently broadcast host presence to specialist herbivores or disrupt the foraging behavior of essential pollinators, with cascading effects on plant reproduction and community structure (Gish et al. 2015). Similarly, root-derived AMPs designed to target soil-borne pathogens can negatively impact non-target beneficial rhizobacteria and mycorrhizal fungi (Murphy et al. 2021; Song et al. 2021). The role of microbiomes is a critical evolutionary component that is often overlooked in engineering strategies. Plants do not evolve in isolation but rather as holobionts, i.e., complex assemblages comprising the host and its associated microbiome (Rosenberg and Zilber-Rosenberg 2018). Engineered changes in root exudates or immune status can selectively enrich or deplete specific microbial taxa, leading to unintended shifts in community structure and function (Berendsen et al. 2018). Such shifts may compromise the natural disease-suppressive capacity of soils, alter nutrient cycling, or even facilitate the emergence of new pathogen pressures. Therefore, a holistic evolutionary perspective must consider the plant as a nexus within an ecological network, where engineered traits can reshape long-standing symbiotic partnerships and ecological community equilibria, with cascading consequences for crop resilience, ecosystem health and agroecosystem sustainability (Rosenberg & Zilber-Rosenberg 2018).

### Challenges in translating microbiome-mediated immunity to the field

While harnessing the plant microbiome for induced resistance holds immense promise, translating this approach into reliable, broad-scale agricultural applications faces significant practical, biological, and ecological hurdles. Inoculated beneficial strains often fail to persist or maintain functionally effective population densities due to intense competition with indigenous microbiota, variability in abiotic factors and heterogeneity in soil chemistry (Toju et al. 2018). This environmental sensitivity leads to unpredictable protection efficacy across different growing seasons and geographical locations, hindering commercial adaptation. The success of microbiome-mediated priming is also highly dependent on the host genotype. The genetic background of plants shapes their rhizodeposition profile, constitutive immune tone, and physical architecture, all of which determine the "cry-for-help" dynamics and colonization success of introduced microbes (Wagner et al. 2020). This specificity necessitates a move away from universal inoculants toward customized microbial consortia or crop varieties specifically bred for increased recruitment and compatibility with key protective microbes. Moreover, introducing microbial agents, especially non-native or engineered strains, carries the risk of unintended ecological consequences. Altering the abundance of specific microbial taxa can have cascading effects on resident community assembly, network interactions, and ecosystem functions. For example, promoting bacterial taxa that prime immunity via the JA pathway might inadvertently suppress competing microbiomes that support SA-mediated defenses. This could alter the defensive capacity of plants against different pathogens (Pieterse et al. 2012; Carrión et al. 2019). Additionally, horizontal gene transfer within engineered or stimulated microbial communities could facilitate the dissemination of antibiotic resistance genes or other traits with unforeseen environmental impacts (Liang et al. 2020). Advancing microbiome engineering toward durable field efficacy requires an integrated framework that combines microbial ecology, systems biology, and rigorous environmental risk assessment. Future strategies must focus on developing resilient, context-adapted microbial consortia and precision delivery systems for sustainable crop protection.

### Technical bottlenecks: bridging the lab-to-field gap

While high-throughput omics technologies provide unprecedented insights, the primary bottleneck lies in their functional integration. While genomics, transcriptomics, proteomics, and metabolomics offer unique insights into different aspects of plant immunity and the development of pathogen-resistant crops, their integration can reveal complex regulatory networks and identify novel targets for improvement (Rajamuthu et al. 2025). The integration of multi-omics datasets requires sophisticated computational approaches, including machine learning algorithms, network analysis methods, and systems biology modeling (Jain et al. 2024). These approaches can identify key regulatory nodes, predict the effects of perturbation, and guide the development of intervention strategies. The development of user-friendly software tools and databases is essential for making these approaches accessible to researchers and breeders.

Bridging the lab-to-field gap also relies on the advancement of high-throughput phenotyping (HTP) platforms (Sheikh et al. 2024). The application of these technologies to disease resistance breeding can accelerate the identification of superior genotypes and improve selection efficiency. The development of precision breeding approaches requires the integration of high-throughput phenotyping with genomic selection and other advanced breeding methods (Visakh et al. 2024). This integration enables the development of predictive models that can guide breeding decisions and optimize selection strategies. The use of artificial intelligence and machine learning algorithms can increase the accuracy of these predictive models and facilitate the identification of complex trait relationships (Sheikh et al. 2024). However, the implementation of high-throughput phenotyping in breeding programs necessitates substantial investments in equipment, training, and data management systems.

Systems and synthetic biology provide a framework for understanding and manipulating complex biological systems, including plant immunity networks (Nayak et al. 2021; Zhang et al. 2025b). These approaches integrate experimental data with computational models to predict system behavior and identify optimal intervention strategies. The application of systems biology to plant immunity can decode emergent properties of defense networks and guide the development of more effective enhancement strategies. However, moving from characterizing individual parts (e.g., promoters and genes) to reliably predicting the behavior of a synthetic circuit in a whole plant remains challenging. The predictive design of synthetic immune circuits is limited by the context-dependence of biological parts and the inherent randomness of living systems. Bridging this predictive gap requires the generation of large, high-quality quantitative data on part performance in planta and the development of more advanced mathematical models that consider the cellular and organismal context.

### Bridging to application: practical and socio-economic hurdles

**Fig. 4:**
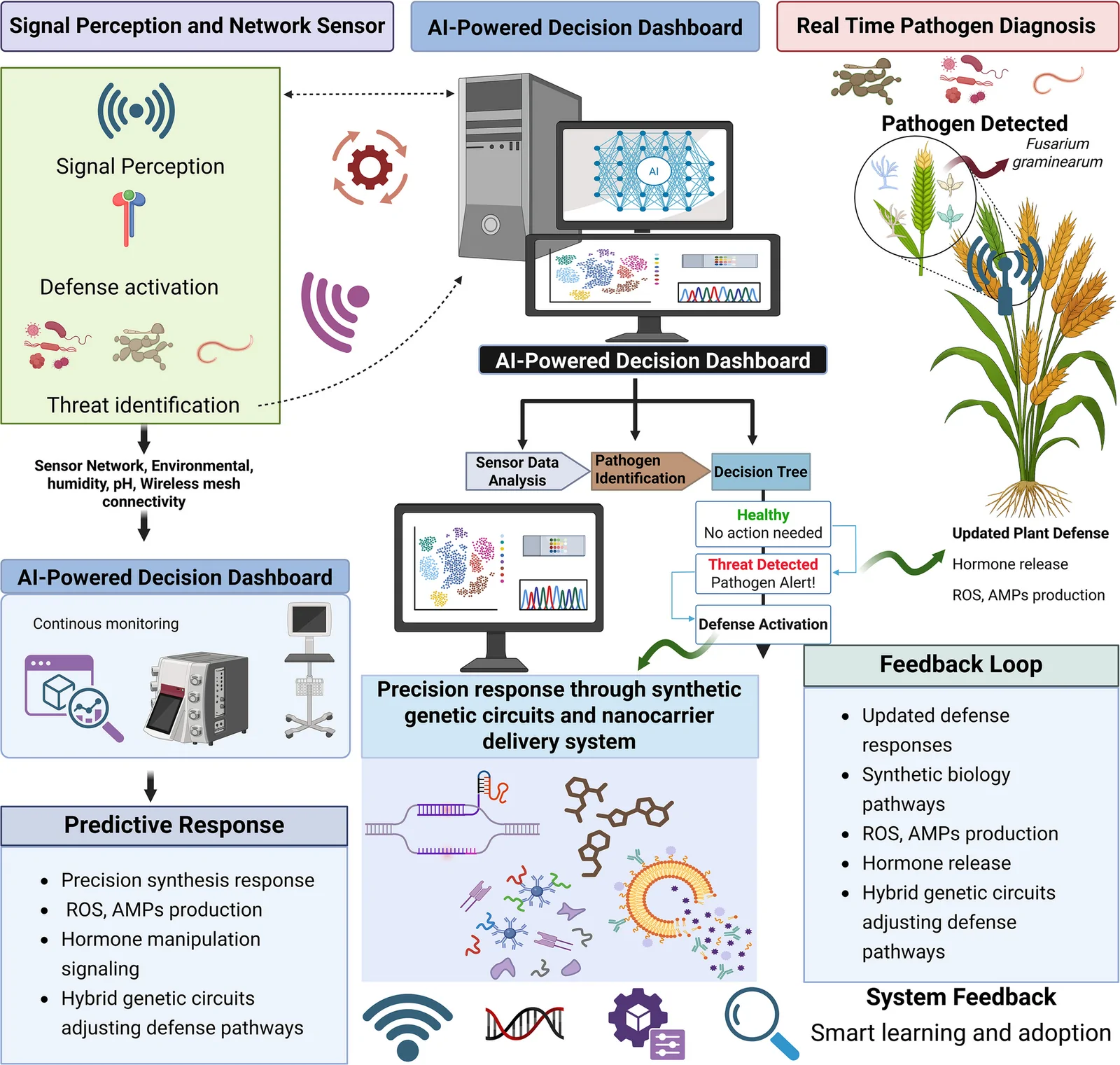
AI-powered plant pathogen detection and defense system: This figure illustrates the integration of a signal perception network, real-time pathogen detection, and AI-driven decision making to activate plant defenses. 1) Diagnosis: Sensors continuously monitor environmental parameters and pathogen-specific signatures. (2) Decision: When a pathogen is identified, an onboard AI module processes and analyzes the data to identify the threat. (3) Precision response: The system activates a targeted, precise response, such as hormone release, ROS and AMPs. The system will also incorporate predictive responses either by activating an engineered, pathogen-inducible genetic circuit or delivering RNAi or elicitors specifically to the infection site. (4) Learning and Reset: A closed feedback loop enhances system performance by fine tuning defense pathways on the basis of pathogen detection and environmental data, optimizing the system's adaptive learning ability and continuously improving plant defense mechanisms

The transition of advanced agricultural technologies from proof-of-concept to widespread field application requires addressing practical, regulatory, and socio-economic barriers. A realistic pathway to adoption must account for long-term environmental safety, evolving regulatory landscapes, manufacturing scalability, and economic viability for end-users. A critical concern is the long-term environmental impact of engineered interventions. NCs can accumulate in the soil ecosystem, potentially affecting non-target soil microbiota, nutrient cycling, and plant health over multiple growing seasons, altering soil chemistry, and impacting plant‒microbe interactions over successive growing seasons (Zhang et al. 2024; Huang et al. 2024; Wang et al. 2024). Similarly, engineered microbial inoculants present ecological and manufacturing challenges. The deliberate release of engineered microbes, whether for immune priming or pathogen exclusion, necessitates comprehensive environmental risk assessment. Key parameters include the potential for horizontal gene transfer, persistence and dispersal beyond the application zone, and impacts on native microbial community structure and function (Jain et al. 2024; Chemla et al. 2025). In addition to these technical challenges, the primary regulatory hurdles for gene-edited crops are complex and lack global regulatory harmonization, creating uncertainty for developers, and potentially delaying access for farmers. Jurisdictions such as the United States, Japan, and Argentina have adopted product-based regulatory frameworks for certain CRISPR‒Cas-edited products lacking foreign DNA, whereas the European Union's process-based approach subjects them to strict GMO regulations. This disharmony complicates international trade and investment. Regulatory clarity, which is grounded in scientific evidence and distinguishes between transgenic and cisgenic edits, is essential for fostering innovation (Vora et al. 2023; Groover et al. 2024; Tuncel et al. 2025). Real-world applications of these advanced technologies are emerging; however, the adoption of advanced technologies largely depends on their economic feasibility for farmers. The cost‒benefit analysis must be compelling, with clear advantages in yield enhancement, input reduction (e.g., pesticides, fertilizers), or resilience to biotic/abiotic stresses. The high costs associated with NCs or microbial formulations, coupled with limited knowledge on application timing and dosage, can restrict their use in high-value crops. Technologies that require significant changes to existing farming practices or infrastructure face additional adoption barriers. Therefore, alongside technical development, parallel efforts in creating supportive extension services, financing mechanisms, and value-chain linkages are essential to ensure that these innovations deliver on their promise of sustainable intensification for farmers of all scales. Figure 4.

### Future directions: toward predictive and climate-resilient immunity

**Fig. 5:**
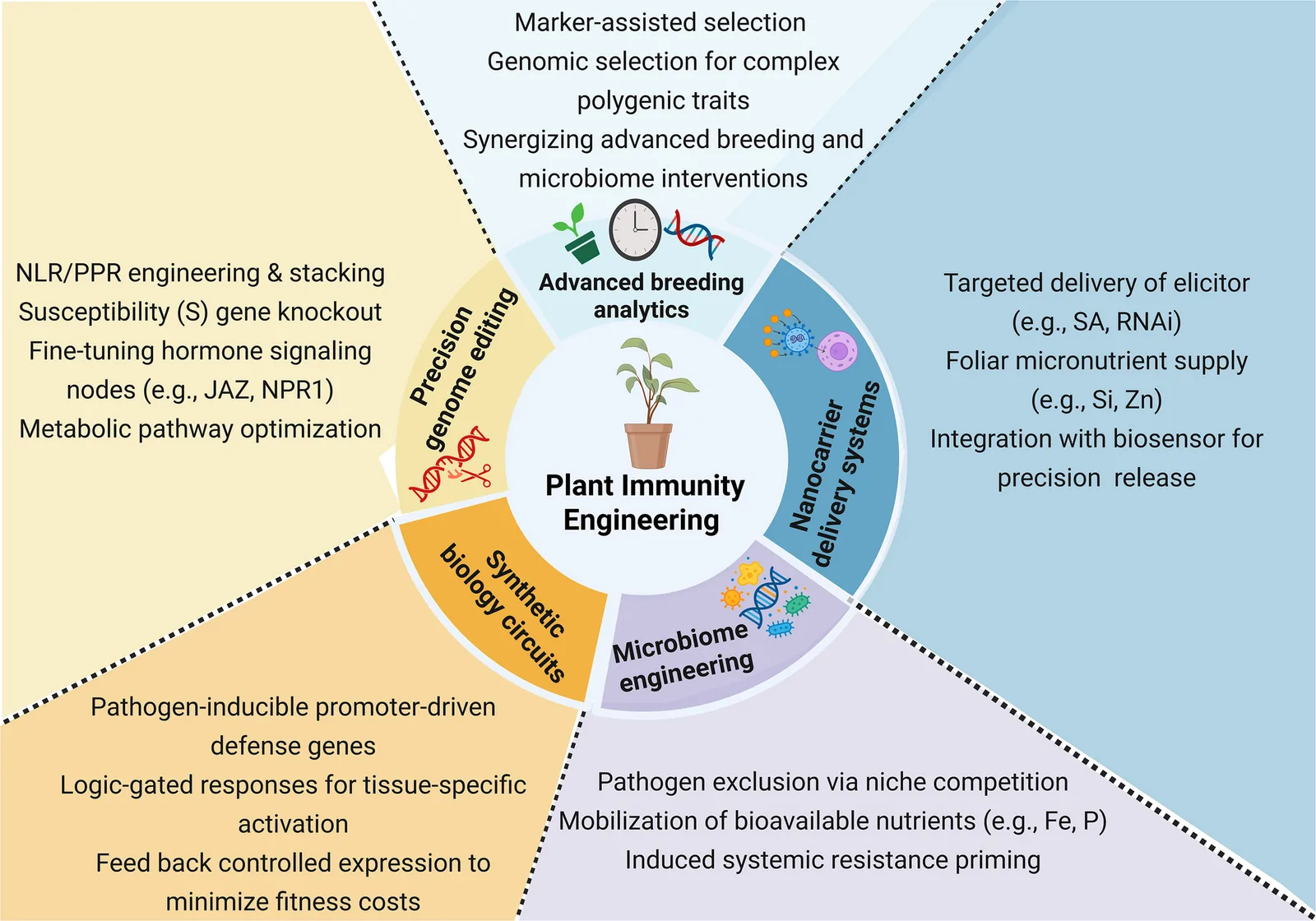
Integrative framework for engineering plant immunity: This schematic highlights the integration of advanced breeding analytics, precision genome editing, synthetic biology circuits, and microbiome engineering to increase plant resilience. Key approaches include marker-assisted and genomic selection for polygenic traits, NLR/PPR stacking, knockout of susceptibility genes, fine-tuning hormone signaling, pathogen inducible promoter-driven defense genes and the use of nanocarrier delivery systems for targeted delivery of elicitors, micronutrients, and biosensor-guided interventions. Additionally, it emphasizes the optimization of metabolic pathways, pathogen exclusion, and the induction of systemic resistance to improve plant resistance against diseases

Despite advances in decoding plant immune signaling and defense metabolism, translating these insights into durable, field-deployable solutions remains a major challenge. Most current knowledge is derived from isolated molecular components or controlled experimental systems, whereas crop protection in agroecosystems requires inducible, systemically coordinated, and context-dependent defense strategies. Future research should therefore develop, and test hypothesis-driven engineering strategies integrated across molecular biology, synthetic biology, nanotechnology, and field agronomy. We propose the following key research questions and a phased development roadmap to guide interdisciplinary efforts. Figure 5.

#### Key research questions

1. Can we develop genetic systems in which enzymes from specific pathogens cleave a synthetic blocker, initiating a series of antimicrobial peptides that stop on their own once the pathogen is gone?
2. How can we carefully design the surface chemistry of nanocarriers to specifically target certain crop species through stomatal tropism while preventing harm to plants and unwanted deposits?
3. Does creating chimeric immune receptors—by combining PRR extracellular domains with NLR‒TIR signaling modules—provide broad resistance without causing constant autoimmunity?
4. Can rhizosphere-competent bacteria engineered to constitutively express plant-derived terpene synthases function as self-sustaining biofumigants that simultaneously prime ISR and directly suppress soil-borne pathogens?
5. Can integrative computational models that use host immune alleles, rhizosphere metagenomes, and soil factors accurately predict disease susceptibility in new environments?

To address these questions, we propose an interdisciplinary roadmap structured around five interconnected stages. 1) Mechanistic discovery through multi-omics analyses across diverse pathosystems. 2) Component design via synthetic biology and materials science. 3) System integration and validation under controlled yet environmentally relevant conditions. 4) Field validation across multiple seasons and agroecological zones to assess durability, yield stability, microbiome dynamics, and ecological compatibility. 5) Translation aligned with regulatory frameworks and agronomic practices, farmer training modules, and extension protocols, prioritizing inducible and microbiome-based solutions where appropriate.

## Data Availability

Not applicable.

## Abbreviations

PTI: Pattern-triggered immunity
ETI: Effector-triggered immunity
SA: Salicylic acid
JA: Jasmonic acid
ET: Ethylene
PPRs: Pattern recognition receptors
PAMPs: Pathogen-associated molecular patterns
ROS: Reactive oxygen species
MAPK: Mitogen-activated protein kinase
NLRs: Leucine-rich repeats
HR: Hypersensitive response
ABA: Abscisic acid
SAR: Systemic acquired resistance
PR: Pathogenesis-related
MeSA: Methyl salicylate
ISR: Induced systemic resistance
PGPR: Plant growth promoting rhizobacteria
BRs: Brassinosteroids
GAs: Gibberellins
CHS: Chalcone synthase
CHI: Chalcone isomerase
FLS: Flavonol synthase
AMPs: Antimicrobial peptides
eATP: Extracellular ATP
eNAD⁺: Extracellular nicotinamide adenine dinucleotide
cADPR: Cyclic ADP‒Ribose
eADP: Extracellular ADP
DAMP: Damage-associated molecular pattern
eCTP: Extracellular CTP
2-dG: 2′-Deoxyguanosine
TIR: Toll/interleukin-1 receptor
JAZ: Jasmonate ZIM-domain
PAL: Phenylalanine ammonia-lyase
NPs: Nanoparticles
RNAi: RNA interference
SENDS: Surface ligand-engineered NPs system
NCs: Nanocarriers
FHB: Fusarium head blight
GS: Genomic selection
QTLs: Quantitative trait loci
PGPF: Plant growth-promoting fungi
BTH: Benzothiadiazole
HTP: High-throughput phenotyping
